# Hypoxia-induced amniotic fluid stem cell secretome augments cardiomyocyte proliferation and enhances cardioprotective effects under hypoxic-ischemic conditions

**DOI:** 10.1038/s41598-020-80326-w

**Published:** 2021-01-08

**Authors:** Marek Kukumberg, Tatsanee Phermthai, Suparat Wichitwiengrat, Xiaoyuan Wang, Subramanian Arjunan, Suet Yen Chong, Chui-Yee Fong, Jiong-Wei Wang, Abdul Jalil Rufaihah, Citra Nurfarah Zaini Mattar

**Affiliations:** 1 Department of Obstetrics and Gynaecology, Yong Loo Lin School of Medicine, National University of Singapore, Singapore, Singapore; 2 Department of Surgery, Yong Loo Lin School of Medicine, National University of Singapore, Singapore, Singapore; 3 Stem Cell Research and Development for Medical Therapy Unit, Department of Obstetrics and Gynecology, Faculty of Medicine Siriraj Hospital, Mahidol University, Bangkok, Thailand; 4 Cardiovascular Research Institute, National University Heart Centre Singapore, Singapore, Singapore; 5 Department of Physiology, Yong Loo Lin School of Medicine, National University of Singapore, Singapore, Singapore; 6 Department of Obstetrics and Gynaecology, National University Health System, Singapore, Singapore

**Keywords:** Stem cells, Pluripotent stem cells, Cardiology

## Abstract

Secretome derived from human amniotic fluid stem cells (AFSC-S) is rich in soluble bioactive factors (SBF) and offers untapped therapeutic potential for regenerative medicine while avoiding putative cell-related complications. Characterization and optimal generation of AFSC-S remains challenging. We hypothesized that modulation of oxygen conditions during AFSC-S generation enriches SBF and confers enhanced regenerative and cardioprotective effects on cardiovascular cells. We collected secretome at 6-hourly intervals up to 30 h following incubation of AFSC in normoxic (21%O_2_, nAFSC-S) and hypoxic (1%O_2_, hAFSC-S) conditions. Proliferation of human adult cardiomyocytes (hCM) and umbilical cord endothelial cells (HUVEC) incubated with nAFSC-S or hAFSC-S were examined following culture in normoxia or hypoxia. Lower AFSC counts and richer protein content in AFSC-S were observed in hypoxia. Characterization of AFSC-S by multiplex immunoassay showed higher concentrations of pro-angiogenic and anti-inflammatory SBF. hCM demonstrated highest proliferation with 30h-hAFSC-S in hypoxic culture. The cardioprotective potential of concentrated 30h-hAFSC-S treatment was demonstrated in a myocardial ischemia–reperfusion injury mouse model by infarct size and cell apoptosis reduction and cell proliferation increase when compared to saline treatment controls. Thus, we project that hypoxic-generated AFSC-S, with higher pro-angiogenic and anti-inflammatory SBF, can be harnessed and refined for tailored regenerative applications in ischemic cardiovascular disease.

## Introduction

Despite significant improvements in cardiovascular research and therapy, the World Health Organization reported that cardiovascular diseases (CVD) are still the leading cause of death globally^1^. Further clinical interrogation of the therapeutic potential of autologous and allogeneic stem cell transplantations in CVD patients, especially of adult-derived stem cells, has shown inconsistent outcomes with no long-term improvement in local and global cardiac function^2–4^. Moreover, the application of embryonic stem cells (ESC) and induced pluripotent stem cells (iPSC), as an alternative to adult-derived stem cells, is hindered by the propensity of ESC toward teratoma formation, and low efficiency and inter-batch variation in generating iPSC^5^. More importantly, FDA regulatory requirements on stem cell therapies further delay these applications^5^. Therefore, the research in this field has diversified to investigating stem cell paracrine effects and the secreted bioactive proteins in such therapies.

Stem cells transplantation in the cardiovascular system has demonstrated limited homing and differentiation within cardiac tissue^6^. However, vascularization of ischemic cardiac tissue was observed, resulting in improved cardiac function^7,8^. Thus, there is speculation that many beneficial effects of stem cell transplantation are due to their modulatory paracrine effects and not directly from transplanted cells^7^. Such results have led to a significant paradigm shift, from exploring stem cell differentiation and tissue regeneration to exploitation of soluble bioactive factors (SBF), characterized as the stem cell secretome (SCS), for functional tissue recovery^9^. The influence of adult-derived SCS on cardiac processes has been demonstrated in various studies^9^. However, adult-derived stem cells are limited by the need for invasive harvest, low yield and varying self-renewal capacity between cell donors. On the other hand, human amniotic fluid stem cells (AFSC) represent a stem cell source, which can be easily isolated by prenatal amniocentesis or at caesarean delivery, is plentiful but regarded as a “reproductive waste” product, yet offers an ideal readily available alternative^10^. AFSC demonstrate properties of both adult-derived and embryonic stem cells but do not form teratomas nor raise ethical concerns over source or derivation. Current research is focused on characterizing the immunophenotype, gene and protein expression of AFSC and on producing enriched secretome^11–13^. AFSC have demonstrated the potential to differentiate into endothelial cells and cardiomyocytes, albeit with limited success^14–18^. The potential of the AFSC secretome (AFSC-S) to influence cellular processes in cardiovascular system is under evaluation^19^. Moreover, the ability to inhibit or enhance cellular processes like apoptosis and proliferation is critical for cardiovascular therapies and a highly sought-after property in SCS^20^. This strategy of targeting of cellular functions via cell-free therapies is desirable in the broader perspective of other diseases that adversely affect endothelial cell and cardiovascular function such as the severe acute respiratory syndrome corona virus 2 (SARS-CoV-2)^21^.

Various approaches have been employed to induce high protein content SCS production, however, no method has been standardized to date^22^. Oxygen modulation is an effective physical method to achieve this, and the effectiveness of hypoxic induction with several stem cell types is well documented^20,23,24^. Such results are encouraging and suggest that SBF concentrations in SCS may be modified for individualized applications. In contrast to production techniques, less is known about the content of most SCS which, as yet, are largely unidentified. To date, no studies have evaluated the SBF secretion dynamics in AFSC-S, the latter’s effects on human adult cardiomyocyte growth and proliferation, or its cardioprotective effect. Here we investigate qualitative and temporal SBF expression in AFSC-S generated in hypoxic and normoxic conditions, its dose-dependent influence on cell proliferation in physiological and ischemic conditions, and its potential in vivo cardioprotective effects in an ischemia/reperfusion (IR) injury animal model.

## Results

### Characterization of amniotic fluid stem cells

**Figure 1:**
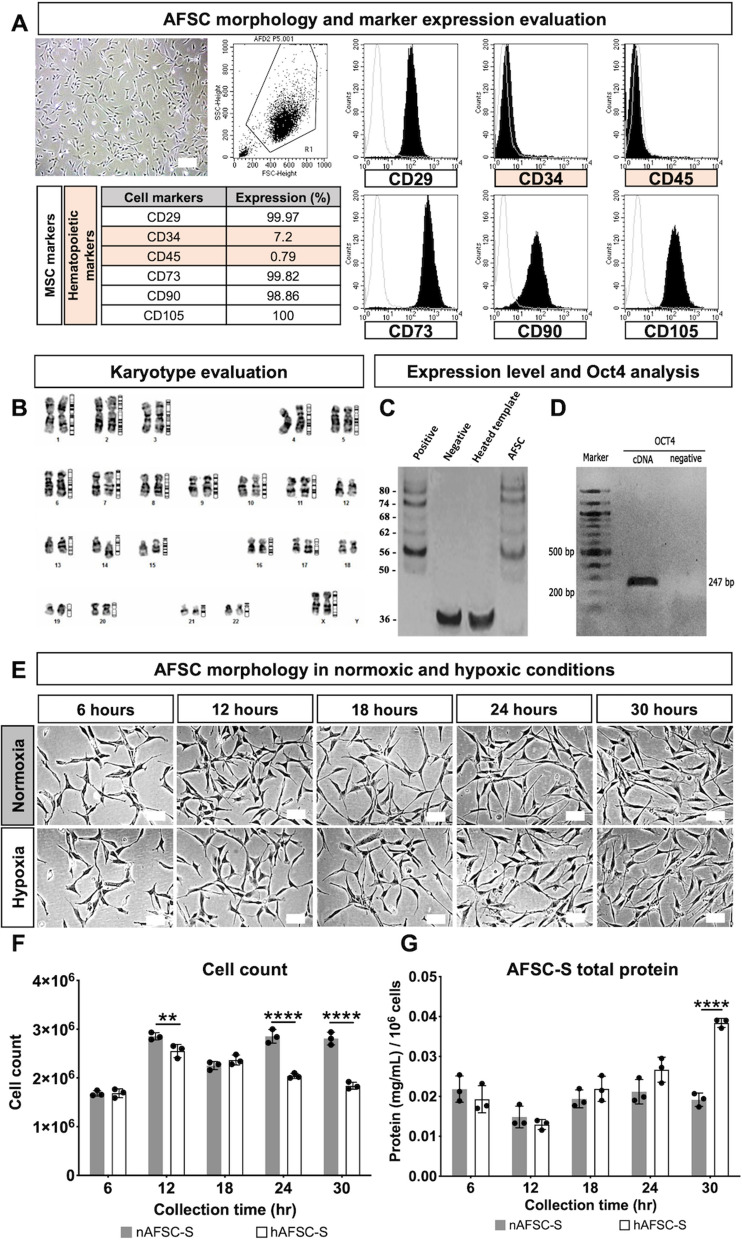
Human amniotic fluid stem cells and amniotic fluid stem cell secretome evaluation. (**A**) Representative image of amniotic fluid stem cells (AFSC) exhibiting fibroblastic morphology and flowcytometric quantification of cell markers. (**B**) Karyotypic analysis. (**C**) Telomerase activity evaluation. (**D**) Oct-4 evaluation. (**E**) Representative images of cell morphology of amniotic fluid stem cells cultured in 21% O_2_ (Normoxia) and 1% O_2_ (Hypoxia) conditions for 6 to 30 h. (**F**) Total count of AFSC. (**G**) Total protein quantification of AFSC secretome (AFSC-S) collected at 6 to 30 h from normoxic (nAFSC-S) and hypoxic (hAFSC-S) culture conditions. Scale bar 100 µm. Data are expressed as means ± SD. n = 3. **p < 0.01, ****p < 0.0001 by two-way ANOVA followed by Tukey’s *post hoc* test.

AFSC clones were generated from primary amniotic fluid cell culture. As reported in our previous studies, clonal AFSC lines derived with the starter cell method showed stable characteristics between lines^11^. Thus, we selected one representative clone for AFSC production. AFSC displayed mean population doubling time of 0.97 ± 0.11 days (range 0.86–1.08 days) through six cultured passages (data not shown), fibroblast morphology and expressed cell surface cluster of differentiation (CD) markers of mesenchymal stem cells, including CD29 (99.9%), CD73 (99.8%), CD90 (98.8%), CD105 (100%), with low expression of hematopoietic markers CD34 (6.9%) and CD45 (0.7%) (Fig. 1A). Conventional G-banded karyotyping showed euploidy of 46XX at subculture passage 4 (Fig. 1B). No clonal numerical or structural cytogenetic alterations were observed. AFSC at passage 4 synthesized telomeric repeats at the 3′-ends of their DNA strands (Fig. 1C) and expressed Oct-4 (247 bp amplicon) (Fig. 1D).

### Composition of human amniotic fluid stem cell secretomes

To investigate the effects of hypoxia on AFSC-S generation we evaluated AFSC morphology, quantity and total secretome protein following normoxic (nAFSC-S) and hypoxic (hAFSC-S) culture. Morphology was normal in both conditions at all time-points (Fig. 1E), but cell counts decreased from 12 h onwards in hypoxia (Fig. 1F), with a trend towards higher protein content in harvested hypoxic hAFSC-S in a time-dependent pattern, significant at 30 h (total protein hAFSC-S 0.35 ± 0.04 mg/mL vs. nAFSC-S 0.26 ± 0.02 mg/mL, *p* < 0.05, Fig. 1G).

**Figure 2:**
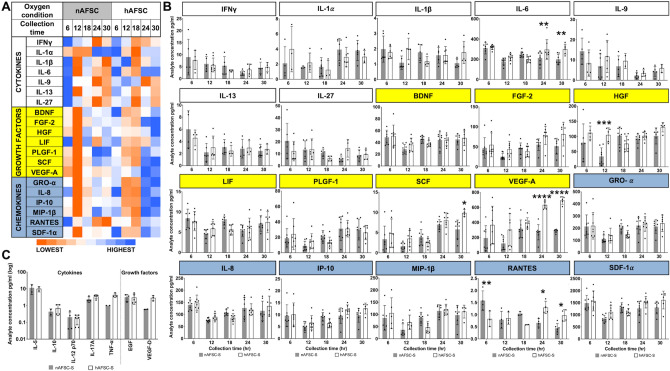
Characterization of all soluble biofactor (SBF) analytes. (**A**) Heat map of all SBF mean values as a comparison of cytokines—Interferon γ (IFN γ), Leukocytic Pyrogen (Interleukin 1β; IL-1β), Hematopoietin 1 (Interleukin 1α; IL-1α), Interleukin 6 (IL-6), Pleiotropic Cytokine (Interleukin 9; IL-9), Interleukin 13 (IL-13), Interleukin 27 (IL-27), growth factors—Brain-derived Neurotrophic Factor (BDNF), Fibroblast Growth Factor 2 (FGF-2), Hepatocyte Growth Factor (HGF), Leukemia Inhibitory Factor (LIF), Placental Growth Factor (PGF), Stem Cell Factor (SCF), Vascular Endothelial Growth Factor A (VEGF-A) and chemokines—Growth Regulated Oncogene α (GRO-α), Neutrophil Chemotactic Factor (Interleukin 8; IL-8), Interferon-γ-inducible Protein 10 (IP-10), Macrophage Inflammatory Protein 1β (MIP-1β), Regulated upon Activation, Normal T Cell Expressed and Presumably Secreted (RANTES) and Stromal Cell-derived Factor 1α (SDF-1α) identified in the human amniotic fluid stem cells secretome (AFSC-S) generated in normoxic (21% O_2_; nAFSC-S) and hypoxic (1% O_2_; hAFSC-S) conditions and collected at 6 to 30 h (Collection time). Lowest and highest values are color labeled (dark orange to dark blue). Heat map evaluation was performed per analyte (rows) with analyte mean values. (**B**) Single SBF analyte concentration comparison in nAFSC-S and hAFSC-S. Values normalized to one million cells. Data are expressed as means ± SD. n = 3–8. (**C**) SBF analyte comparison detected at 12 h only. Values normalized to one million cells and presented as log of 10 ± SD. n = 2–6. *p < 0.05, **p < 0.01, ***p < 0.001, ****p < 0.0001 by two-way ANOVA followed by Tukey’s post hoc test.

Twenty-seven SBF were identified in both secretomes in varying concentrations (Table 1A). Mean concentrations of individual analytes produced in secretomes are represented as a heatmap showing the temporal change in analyte concentrations (Fig. 2A). Overall, the highest concentrations of most growth factors (FGF-2, HGF, LIF, PLGF-1, SCF, VEGF-A) and all chemokines (GRO-α, IL-8, IP-10, MIP-1β, RANTES and SDF-1α) were observed in hAFSC-S collected at 24 h and 30 h, while a concomitant reduction in cytokines IFN-γ, IL-9, IL-13, IL-27 was seen at the same time-points. Conversely, the highest cytokine concentration occurred at 6 h regardless of oxygen condition (Fig. 2B). The lowest concentrations of most SBF were observed at 12 h (in nAFSC-S) and 18 h (in hAFSC-S). There was a higher expression of growth factors and chemokines than cytokines at all time-points. IL-6 (318.07 ± 22.34 pg/ml), VEGF-A (693.33 ± 51.91 pg/ml), SDF-1α (1,574.65 ± 411.86 pg/ml) were present in the highest concentrations of all cytokines, growth factors and chemokines respectively, and IL-1β, IL-1α, SCF and RANTES were least expressed (Fig. 2B). Analyte concentrations remained stable, ranging from 0.43 pg/ml (nAFSC-S, RANTES, 30 h) to 1574 pg/ml (hAFSC-S, SDF-1α, 6 h). Limited data was available for seven analytes (IL-5, IL-10, IL-12 p70, IL-17A, TNFα, EGF, VEGF-D), which showed no differences at 12 h between nAFSC-S and hAFSC-S (Fig. 2C).

**Table 1:**
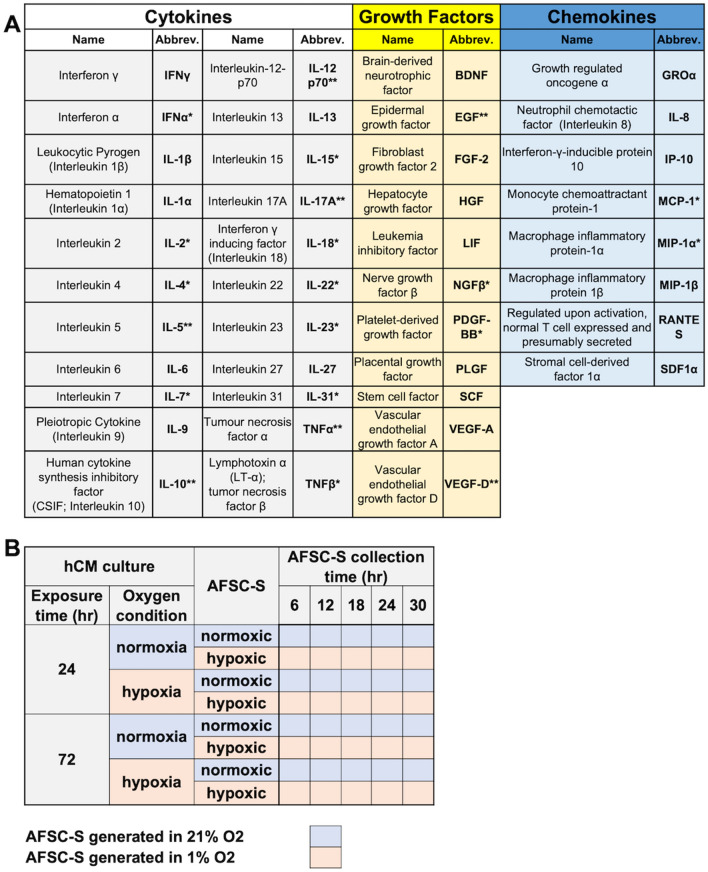
List of analytes and secretomes.

A) List of all 41 screened analytes. Analytes grouped according to their functions as cytokines, growth factors and chemokines. Not detected analytes—one asterisk, analytes compared only in 12 h-nAFSC-S and 12 h-hASFC-S—two asterisks. B) List of AFSC-S and hCM culture conditions. Respective abbreviations indicated in both tables.

### AFSC-S promotes human cardiomyocyte proliferation

**Figure 3:**
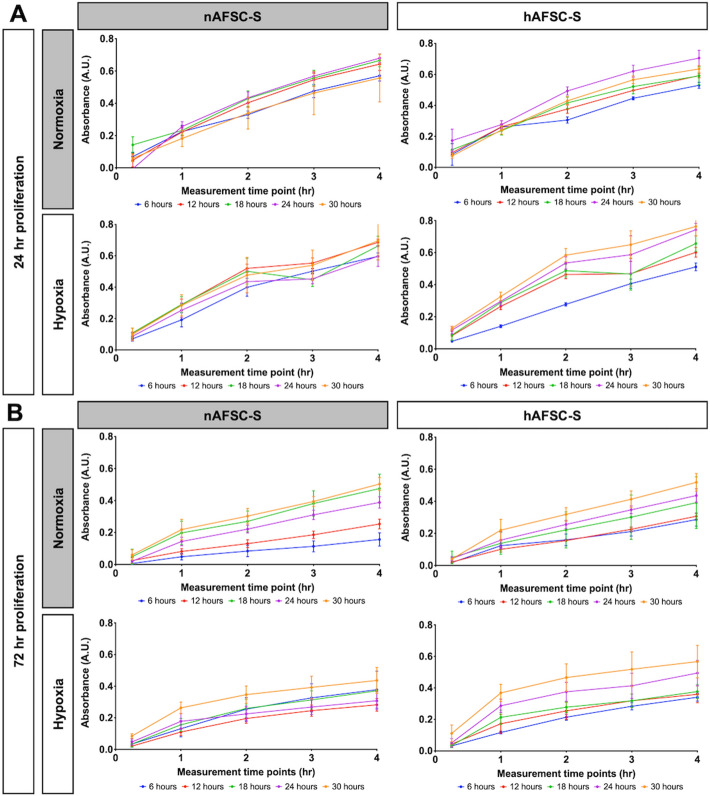
Evaluation of the temporal growth curve of human cardiomyocytes (hCM). Comparison of the MTS tetrazolium compound reduction in a colorimetric assay to asses proliferation of hCM cultured for (**A**) 24 h (24 h proliferation) and (**B**) 72 h (72 h proliferation) in 21% O_2_ (Normoxia) and 1% O_2_ (Hypoxia) culture conditions with normoxic (nAFSC-S) or hypoxic (hAFSC-S) amniotic fluid stem cells secretome collected at 6 (blue), 12 (red), 18 (green), 24 (purple) and 30 h (orange). The first absorbance (A.U.) measurement was recorded at 15 min and then subsequently at 1, 2, 3 and 4 h. Date are expressed as means ± SD. n = 5.

**Figure 4:**
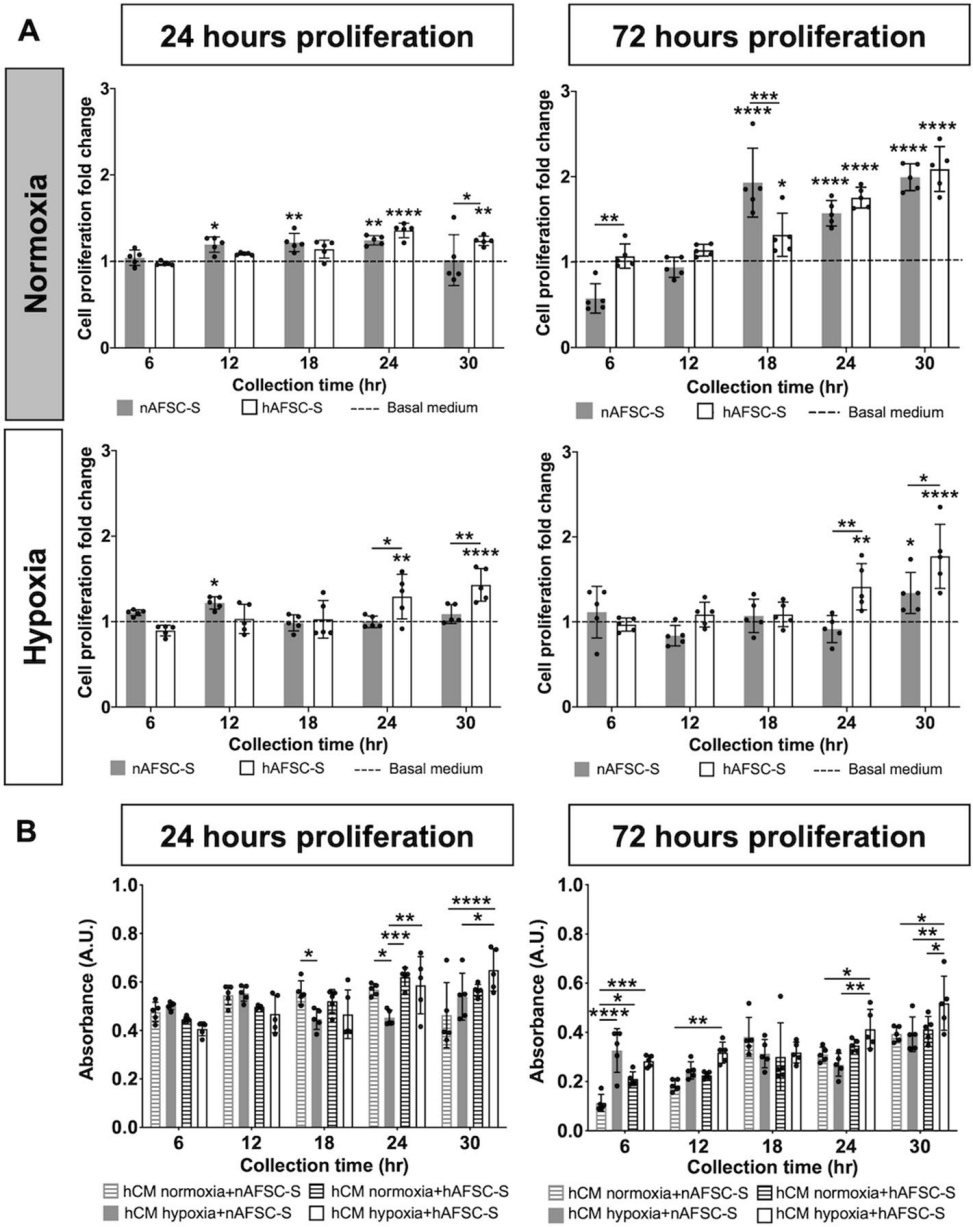
Comparison of human cardiomyocyte (hCM) proliferation. Comparison of the MTS tetrazolium compound reduction in a colorimetric assay to asses hCM proliferation cultured for 24 or 72 h in normoxic (Normoxia) and hypoxic (Hypoxia) culture conditions with amniotic fluid stem cells secretome (AFSC-S) collected from normoxic (nAFSC-S) or hypoxic (hAFSC-S) conditions collected at 6 to 30 h. (**A**) Cell proliferation fold change evaluation of recorded values normalized to hCM proliferation values in control basal medium (basal MEMα; dashed line) and (**B**) absolute absorbance values cross parameter comparison. Data are expressed as means ± SD. n = 5. * above a bar graph indicates statistical significance between AFSC-S group and basal MEMα group. * above a line indicates statistical significance between nAFSC-S and hAFSC-S group. *p < 0.05, **p < 0.01, ***p < 0.001, ****p < 0.0001 by two-way ANOVA followed by Tukey’s *post hoc* test.

To investigate human cardiomyocytes (hCM) proliferation following exposure to nAFSC-S or hAFSC-S, cultures were performed for 24 h and 72 h in normoxia and hypoxia (Table 1B), following which continued growth was observed during the MTS proliferation assay (Fig. 3). Higher absorbance (indicative of active cell proliferation) was observed after 24 h in all conditions (Fig. 3A). A larger variation in cell proliferation between individual AFSC-S time points was observed after 72 h in culture with nAFSC-S and hAFSC-S (Fig. 3B). hCM displayed higher proliferation with either secretome in normoxia and hypoxia over hCM cultured in basal MEMα medium (control, represented by dashed line, Fig. 4A), significant with AFSC-S collected at 24 h and 30 h. To simulate prolonged ischemic conditions *in vivo*, we exposed hCM to a 72 h cell culture in hypoxia and compared it to hCM cultured for 72 h in normoxia with nAFSC-S and hAFSC-S. Similar to 24 h AFSC-S exposure, hCM proliferation in secretome was higher than hCM proliferation in basal medium, particularly with hAFSC-S harvested at the predetermined collection points from 18 h onwards (Fig. 4A). Cross-parameter evaluation demonstrated higher cell proliferation following 24 h of culture using AFSC-S collected at 24 h and 30 h, in hypoxia, with hAFSC generally performing better than nAFSC (Fig. 4B).

### The influence of AFSC-S on cell growth is dependent on dose and cell type

**Figure 5:**
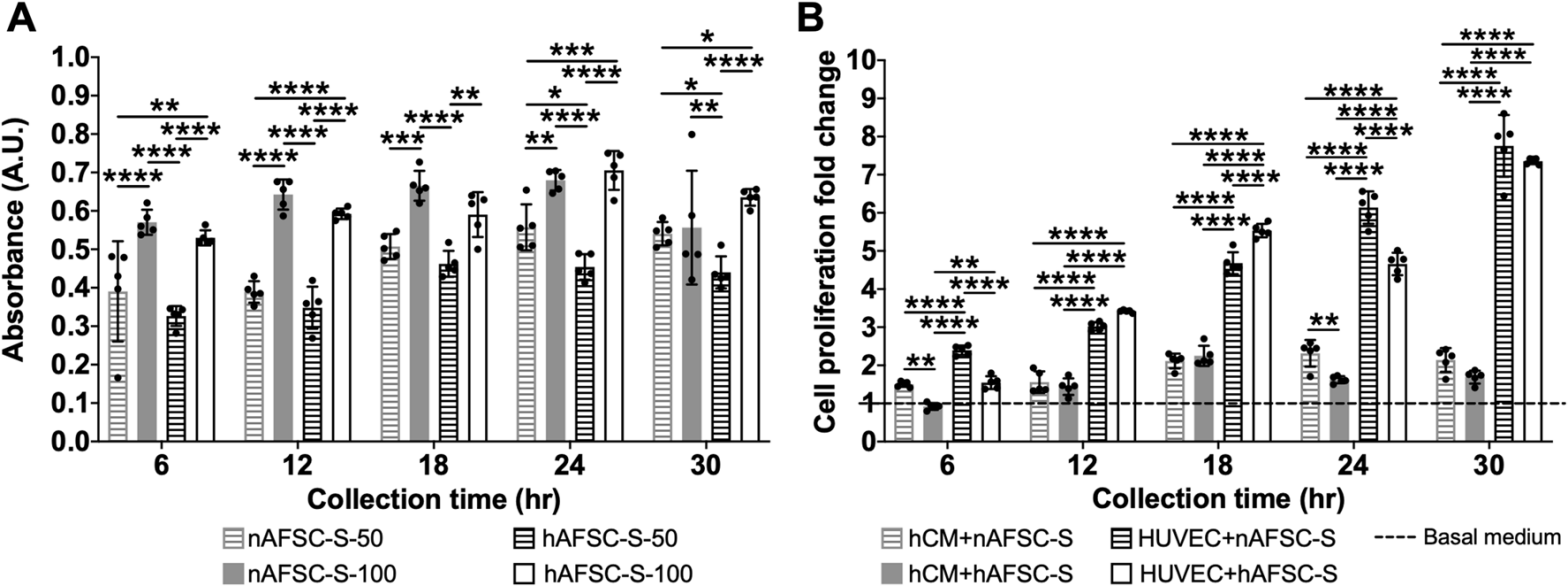
Dose-dependent and cell type influence proliferation. Comparison of the MTS tetrazolium compound reduction in a colorimetric assay to assess proliferation of cells cultured for 24 h. Cells cultured in human amniotic fluid stem cells secretome cultured in normoxic (21% O_2_, nAFSC-S) or hypoxic (1% O_2_, hAFSC-S) conditions and collected at 6 to 30 h. (**A**) hCM cultured in 50% (nAFSC-S-50 or hAFSC-S-50) or 100% (nAFSC-S-100 or hAFSC-S-100) secretome dose. (**B**) Cell proliferation presented as fold change of human cardiomyocytes (hCM) and human umbilical vein endothelial cells (HUVEC) proliferation normalized to cell proliferation in basal media (dashed line). Data are expressed as means ± SD. n = 5. *p < 0.05, **p < 0.01, ***p < 0.001, ****p < 0.0001 by two-way ANOVA followed by Tukey’s *post hoc* test.

To determine dose dependency of AFSC-S on hCM proliferation, we cultured hCM in 50% and 100% nAFSC-S or hAFSC-S for 24 h in normoxia. Higher cell proliferation was observed with 100% nAFSC-S and hAFSC-S (v. 50% AFSC-S) at all time-points (Fig. 5A). We performed a similar interrogation using human umbilical cord endothelial cells (HUVEC) to assess AFSC-S effects on other cardiovascular cell types. HUVEC proliferation was significantly enhanced following 24 h culture in nAFSC-S and hAFSC-S in normoxia compared to basal media culture (control, dashed line, Fig. 5B), with fold-change ranging from 2.40 ± 0.12 to 7.76 ± 0.81 in nAFSC-S and 1.55 ± 0.17–7.36 ± 0.08 in hAFSC-S, higher than hCM proliferation under the same conditions (fold-change of 1.51 ± 0.07–2.14 ± 0.31 in nAFSC-S and 0.92 ± 0.09–1.69 ± 0.17 in hAFSC-, *p* < 0.0001). HUVEC proliferation increased steadily in culture with nAFSC-S and hAFSC-S harvested at all time-points from 6 to 30 h, peaking with 30 h secretomes. Conversely, hCM showed similar cell proliferation with both AFSC-S collected at all time-points (Fig. 5B).

### AFSC-S reduces infarct size in a mouse model of ischemia/reperfusion injury

**Figure 6:**
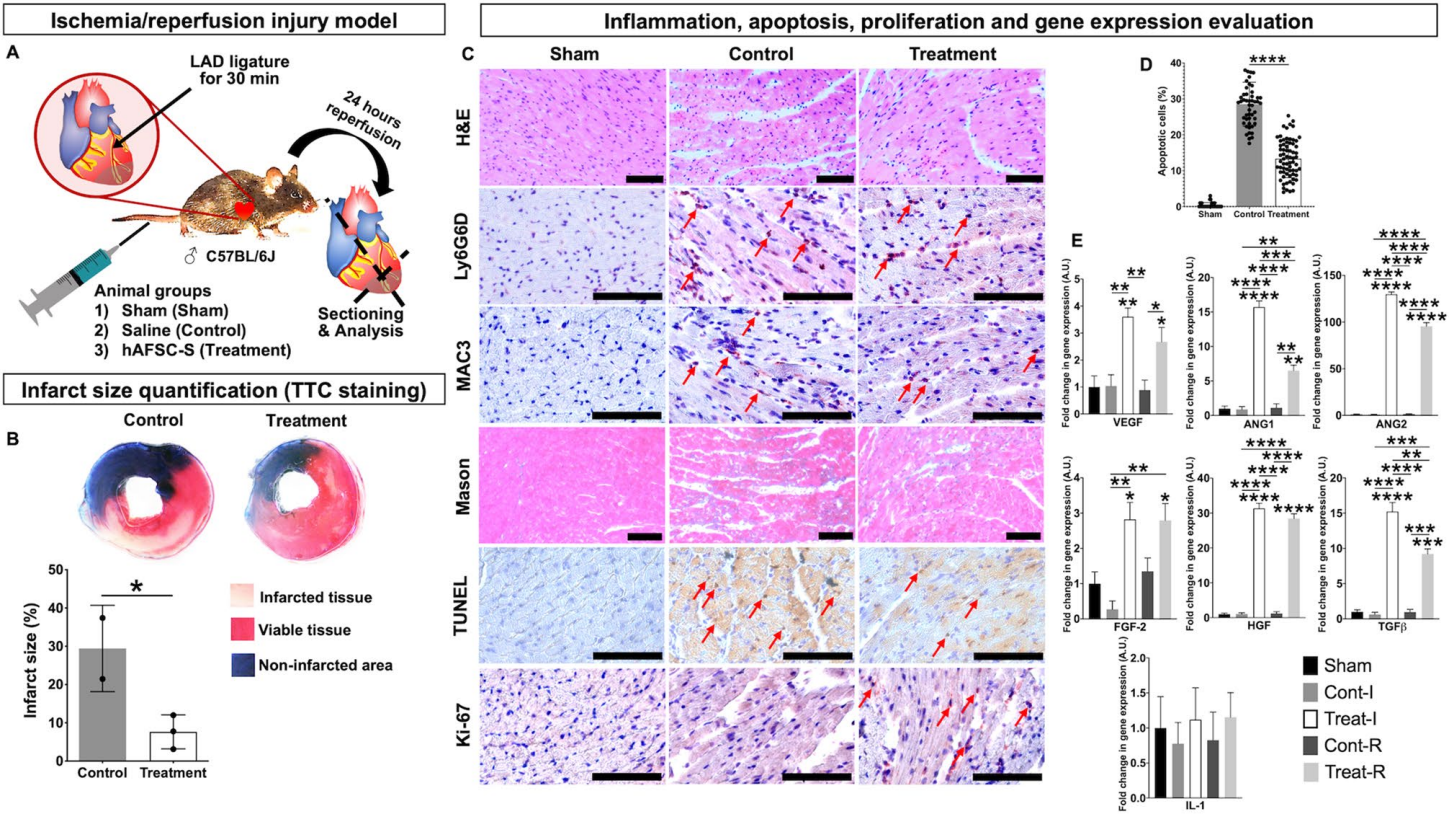
Human hypoxic amniotic fluid stem cells secretome (hAFSC-S) reduces myocardial tissue injury in an ischemia/reperfusion (IR) injury mouse model. (**A**) Male C57BL/6 J mice were treated with 1) saline (Control, n = 4), 2) 20x  concentrated 30h-hAFSC-S (Treatment, n = 6) injected via tail vein 5 min before removing the vascular occlusion. One mouse was used as a sham. Animals were sacrificed after 24 h of reperfusion. (**B**) The mouse heart (saline n = 2, treatment n = 3) was cross-sectioned into 5 pieces and stained in 1%TTC (triphenyl tetrazolium chloride). The non-infarcted area was stained in blue (Evan’s blue; Non-infarcted area). Within the area at risk, infarcted tissue was stained in pale pink (Infarcted tissue) and viable tissue in red (Viable tissue). Infarct size was calculated as the percentage of Infarcted tissue of ischemia-affected tissue. Data are expressed as means ± SD. *p < 0.05 by unpaired t-test. (**C**) Longitudinal heart sections (sham n = 1, saline n = 2, treatment n = 3) were stained for 1) tissue composition (hematoxylin–eosin, H&E), 2) neutrophils (Lymphocyte antigen 6 complex locus G6D, Ly6G6D), 3) macrophages (macrophage differentiation antigen, MAC3), 4) collagen deposition (Masson Trichome), 5) cell apoptosis (Terminal deoxynucleotidyl transferase dUTP nick end labeling, TUNEL) and 6) cells in active phases of cell-cycle (Ki-67). Red arrows indicate positively stained cells. Scale bar 100 μm. (**D**) Apoptotic cells quantification expressed as percentage of TUNEL positive cells over total amount of cells. Data are expressed as means ± SD, ****p < 0.0001 by one-way ANOVA. E) Quantitative real-time polymerase chain reaction (qRT-PCR) analysis of pro-angiogenic (VEGF, ANG1, ANG2, FGF-2, HGF), anti- (TGF-β) and pro-inflammatory (IL-1) genes in ischemic (I) and remote (R) areas of heart tissue in sham (Sham), saline control (Cont-I, Cont-R) and 30h-hAFSC-S treatment (Treat-I, Treat-R). Beta-actin (β-Actin) was used as the normalization control and sham control sample were used as the calibrator. The relative quantification was performed using the comparative CT (2−ΔΔCT) method. * above the bar indicates statistical significance between sham and saline (Cont-I, Cont-R) or treatment groups (Treat-I, Treat-R). * indicates statistical significance between saline (Cont-I, Cont-R) and treatment (Treat-I, Treat-R). Data are expressed as means ± SD. *p < 0.05, **p < 0.01, ***p < 0.001, ****p < 0.0001 by two-way ANOVA followed by Tukey’s *post hoc* test.

To evaluate the *in vivo* cardioprotective potential of hAFSC-S on hCM in both ischemic and reperfusion environments, we systematically administered secretome to the ischemia/reperfusion (IR) injury model. We selected the 30h-hAFSC-S that demonstrated optimal *in vitro* efficacy to treat 10–14 month old mice (~ 38–47 human years^25^), simulating reduced cardiac regenerative abilities in older recipients^26^. Mice received 20x concentrated 30h-hAFSC-S (n = 6) or saline (n = 4) and were sacrificed after 24 h (Fig. 6A). The sham (n = 1) received no injection. No obvious adverse effects were observed during or following injection (e.g. toxicity, anaphylactic shock). The pre-treatment ischemic area in the heart following left anterior descending coronary artery (LAD) occlusion was similar between saline and 30h-hAFSC-S groups (50.1 ± 0.7% vs. 49.5 ± 8.1% respectively, *p* = 0.80). Cardiac infarct size, presented as percentage of the area at risk, was smaller in 30h-hAFSC-S treated mice than infarcts of saline-treated controls (7.6 ± 4.0% vs. 29.1 ± 11.0% respectively, *p* = 0.02, Fig. 6B), representing an ~ 85% vs. 41.2% infarct size reduction. Notably, although 30h-hAFSC-S was unable to reverse ischemic injury entirely, mice treated with 30h-hAFSC-S showed reduced cardiomyocyte separation and inflammatory cell infiltrate within tissue fractures (H&E, Masson Trichome, IHC), reduced apoptosis (TUNEL) and increased cell proliferation (Ki-67) compared to saline and sham controls (Fig. 6C). Apoptotic cells were significantly decreased in 30h-hAFSC-S treated mice compared with saline controls (13.3 ± 5.3 vs. 28.5 ± 6.1% respectively, *p* < 0.0001, Fig. 6D). Mice treated with saline or 30h-hAFSC-S showed neutrophil and macrophage infiltration (Ly6G6D and MAC3 staining respectively, Fig. 6C). Ischemic (Treat-I) and remote areas (Treat-R) both showed significantly upregulated angiogenesis (VEGF, ANGPTL1, ANGPTL2, FGF-2, HGF) and anti-inflammatory genes (TGF-β) with 30h-AFSC-S compared to sham and saline (Cont-I, Cont-R) controls, with no significant difference in pro-inflammatory IL-1 expression (Fig. 6E). Fold increases in gene expression ranged from 2.67 to 129.85, significantly higher than sham and saline controls (Fig. 6E).

## Discussion

The main disadvantages of adult-derived stem cells as therapeutic agents are limited differentiation capability (restricted to multipotency), low isolation yield and difficulty in generating large cell quantities in culture^10^. AFSC are relatively primitive extra-fetal stem cells which have highly proliferative and pluripotent characteristics, comparable to other early developmental stem cells from umbilical cord and placenta, and phenotype and functional expression are similar to mesenchymal and embryonic stem cells^13^. However, homing, survival and differentiation capabilities of AFSC, like stem cells from other sources, are limited^18, 27^. We have previously evaluated AFSC lines from various donors and demonstrated comparable stem cell characteristics, including cell morphology, pluripotent markers (Oct4, SSEA-4), cell surface protein markers (CD29, CD34, CD44, CD45, CD73, CD90, CD105), proliferation ability and multilineage differentiation into endodermal, mesodermal and ectodermal tissues^28,29^. For the current study we randomly selected three AFSC donors from our biobank of over 30 cell lines and evaluated the secretome characteristics (data not shown). As all analytes were comparable and demonstrated similarities in AFSC secretomes, one donor sample was randomly selected and utilized for the rest of the study^30–34^. To further verify the universality of AFSC secretomes and their therapeutic potential, a broader range of donor AFSC should be studied.

SCS promote a substantial regenerative effect, and when generated by reproductive stem cells, produce 10 × to 100 × higher SBF concentrations, showing more pronounced therapeutic effects than SCS generated by adult stem cells^35^. SBF released in the AFSC secretome confer immune-modulatory, anti-inflammatory and regenerative properties via the paracrine effect that could be beneficial in cardiovascular applications, and AFSC-S induce dose-dependent angiogenesis and vasculogenesis in ischemic animal models^36–38^. Several SBF were identified as cytokines, such as interleukins IL-10, IL-1ra, IL-13 and IL-27, which play important roles in local and systemic down-regulation of pro-inflammatory mediators^37^. Other identified SBF such as OPN, MIG and GDF-15 play critical roles in cardiac remodeling and homeostasis^39,40^. The co-culturing of stem cells, stem cell-biomaterial combinations, genetic manipulations of stem cells and preconditioning with biochemical and physical cues have been previously explored to “boost” the release of paracrine factors within the SCS^19,20,23,27^. SCS also contains extracellular vesicles (EVs) of various sizes that carry different ribonucleic acids (RNA), deoxyribonucleic acids (DNA), lipids, metabolites and proteins^41^. EVs are critical for cell-to-cell communication, immunomodulation, SBF transfer and other physiological and pathological processes in several organ systems, including the CVS^41–44^. Previous studies demonstrated that with enrichment, concentration and isolation, EVs may present a therapeutic advantage over other drug applications^20,43–46^. Moreover, even small doses of preconditioning cues can modulate the relative quantities of proteins and extracellular vesicles (EVs) released by stem cells^23^.

Modifying oxygen concentration during cell culture induces up to two-fold higher SBF production, and SBF-enriched hypoxic secretome significantly enhanced vasculogenesis in various tissues *in vitro*^20,23^. Our findings demonstrate that oxygen modulation during secretome generation can be used to upregulate certain SBF and may be a useful way of producing secretome tailored for individualized applications. We have identified a broad range of cytokines, growth factors and chemokines in AFSC-S, where the majority of identified SBF are linked to vasculogenesis pathways. IL-6, VEGF, SDF-1α, GRO-α, IL-8, IP-10 and MIP-1β are interlinked and involved in inflammatory mediation and angiogenic proliferation in post-ischemic conditions such as acute myocardial infarction (AMI) and chronic limb ischemia (CLI)^47–50^. VEGF-A, SDF-1α, GRO-α play further pivotal roles in immunomodulation by regulating activation response, migration, maturation and differentiation of dendritic cells and various T-cell subsets including natural killer T cells^47–50^. Although IL-6 has been described as a myocardial fibrosis promoter, it also has significant capacity to modulate murine neonatal cardiomyocyte proliferation by enhancing relevant protein expression and subsequently promoting cardiac regeneration^51–53^. Growth factor VEGF-A is a crucial pro-angiogenic agent in cardiac repair as a potent inducer of endothelial proliferation, and critically influences migration, survival and hematopoiesis regulation^50^. Indeed, our data suggest that endothelial cells are more susceptible to secretome containing VEGF-A, showing significantly higher proliferation than hCM. Chemokines identified in AFSC-S also exhibit cardioprotective proliferation-inducing properties observed in cardiomyocytes and endothelial cells^39,49,54^. The observed positive influence on cardiomyocytes *in vitro* and *in vivo* may well result from a combined additive and/or synergistic influence of SBF and EVs; a detailed study will be necessary to characterize the influence of AFSC EVs.

Terminally-differentiated human adult cardiomyocytes are post-mitotic cells with limited proliferation, but are still capable of self-renewal^55^. Although several reports have indicated a horizontal transfer of genetic material including mitochondrial DNA from EVs into recipient cells, resulting in increased host cell metabolic activity, other studies describe multiple ways by which cardiomyocytes reactivate endogenous mechanisms to re-enter the cell-cycle and increase cell proliferation, regeneration and renewal^43,44,56–59^. The MTS assay used in the current study permitted colorimetric evaluation of multiple parameters as a high-throughput indicator of proliferation and cell survival to identify the most efficient secretome for further evaluation. We speculate that various factors in AFSC-S, including SBF and EVs, induce one or multiple cardiomyocyte pathways to re-activate cell proliferation. Moreover, hypoxia may contribute to compositional changes of AFSC-S and hence influence cardiomyocyte cell-cycle reactivation *in vitro*^31,60^. However, a more detailed evaluation of re-activated cellular pathways *in vitro* and *in vivo* is necessary to answer this question.

The proliferation and survival of cardiomyocytes and vascular cells is regulated by various signaling pathways such as the PI3K-Akt pathway, activated following release of proteins such as VEGF, RANTES, IL-8 and SDF-1α^61^, demonstrating anti-apoptotic function which significantly increases during ischemia, evidenced by enhanced phosphorylation of Akt in ischemia animal models. Equally important is the ERK1/2 pathway activated by growth factor stimulation and integrin clustering^62^. Only inhibition of ERK1/2 increases cell apoptosis, suggesting its participation in cardiomyocyte and endothelial cell survival^61^. In line with this concept, we postulate that adult hCM exposed to stress conditions, such as ischemia, have limited capability to produce the necessary stimulants to activate these signaling pathways. Therefore, a therapeutic approach focused on augmenting stimulation of affected cells with critical cytokines, chemokines and growth factors will be greatly beneficial. Our data suggests that AFSC-S provides sufficient support for cell proliferation even in prolonged 72 h culture, regardless of cell culture oxygen conditions, in a dose-dependent manner as demonstrated previously^38^. Cell proliferation did not progress in a linear manner over the limited observational period and the different contributary pathways require further evaluation.

**Figure 7:**
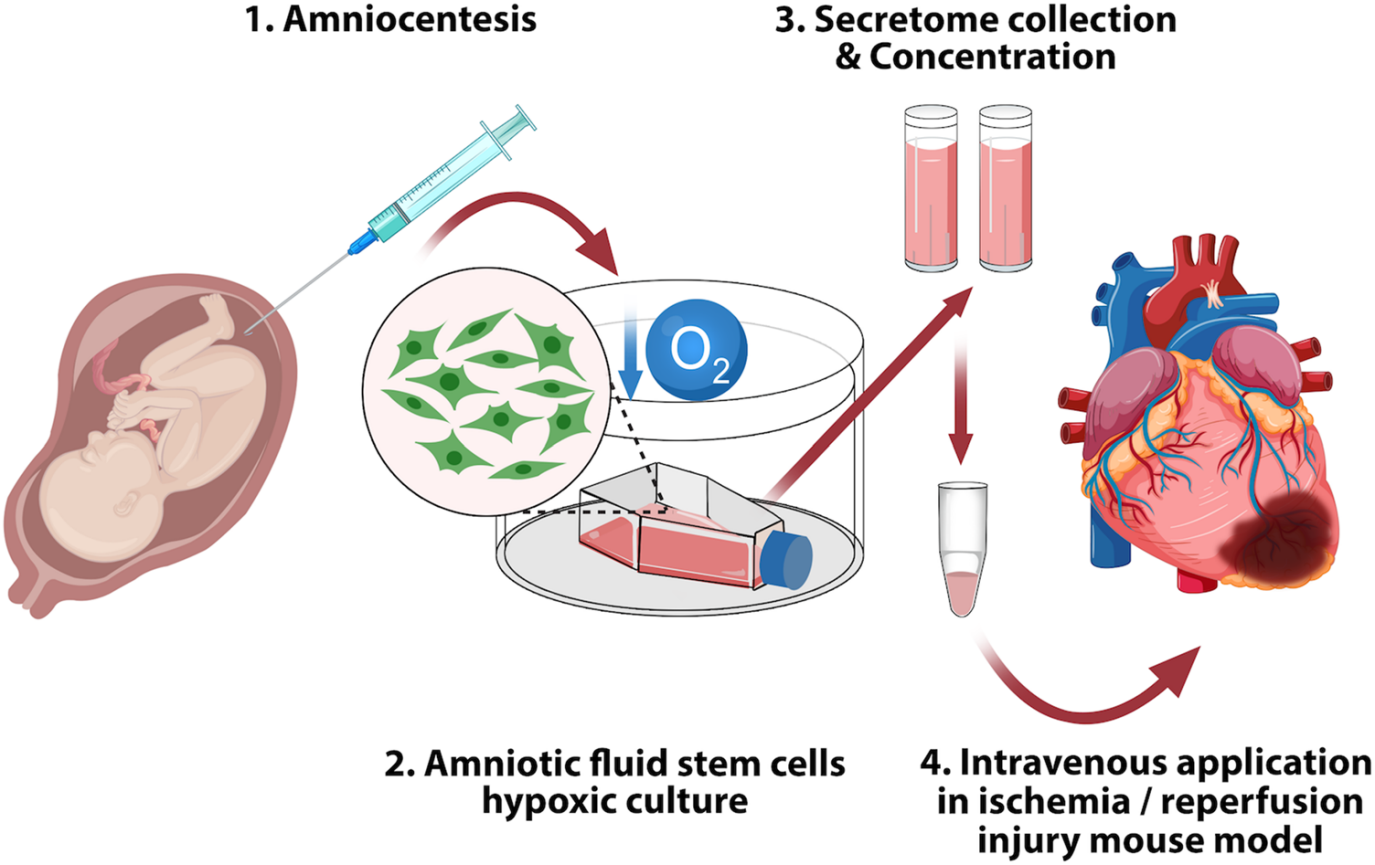
Graphical schematic of experimental approach.

As the use of SCS or its components may be advantageous over stem cell transplantation and our data demonstrated enhanced hCM growth in hAFSC-S, we evaluated AFSC-S in an animal model. Although, surgical interventions post-AMI can restore blood flow, reperfusion carries the risk of additional endothelial-myocardial impairment and increased susceptibility of cardiomyocytes to injury^63–65^. Clinical translation of cardioprotective treatments verified in animal models of IR injury has been unsatisfactory^66^. Several studies have demonstrated the involvement of numerous pathways that lead to functional damage and apoptosis of various cell types including cardiomyocytes, smooth muscle and endothelial cells during cardiac ischemia^54,61,62,66^. Although single-agent therapies have shown success in some patients, the response of other patients with comorbidities to single and end-target therapies has been poor. Consequently, multitargeted therapies (additive or synergistic) may be required for optimal cardioprotection critical for cell survival and the preservation of myocardial hemodynamic performance^54,66^. We postulated that application of an enriched complex SBF secretome may enhance cardioprotection. A recent study demonstrated the application potential of AFSC-S in an ischemic mouse model, albeit non-dose dependent and directly injected into the ischemic cardiac tissue^43^. Myocardial ischemia causes irreversible damage to hCM, and reperfusion risks additional cardiac damage^63–65^. We demonstrated that a single peripheral venous administration of AFSC-S significantly reduces the cardiac infarct area in the IR injury mouse model, possibly through the upregulation of pro-angiogenic and anti-inflammatory genes, reduction of apoptosis and inflammation, and enhanced cardiomyocyte metabolic activity and proliferation (Fig. 7). Our model was made more representative with the use of older adult male mice. Further investigations are required to parse the mechanisms of this effect. Additionally, studying other models of ischemia such as stroke and peripheral arterial disease would broaden the application potential of oxygen-modulated AFSC-S.

The bioengineered SBF composition, dose-specific scale-up and acellular character represent clear therapeutic benefits of SCS, in addition to efficient production, storage and transportation^67^. However, to date there are no standard protocols for SCS bioprocessing^27,67^. Our study demonstrates that oxygen modulation can be an efficient physical cue to induce SBF-enriched SCS for cardiac applications. A multifactorial approach to SCS modulation simulating the complex symbiotic influences in the stem cells microenvironment may further enhance the ability to personalize this therapy.

### Study limitations

This study provides insight into the composition and effect of SBF on cell proliferation *in vitro* and cardiac protection in vivo but granular studies of cellular mechanisms have not yet been performed. A more detailed characterization of angiogenic, apoptotic and inflammatory factors and pathways, and interaction with other components including EVs, is necessary to parse the mechanisms of AFSC-S effects on cardiac remodeling and regeneration.

## Material and methods

### Oxygen modulation

Cells were cultured in normoxia (21% O_2_) in a regular CO_2_ incubator or in hypoxia (1% O_2_) in a hypoxic chamber (Hypoxia Incubator Subchamber, BioSpherix, Parish, NY, USA).

### Human amniotic fluid stem cells (AFSC) culture

The study was conducted in accordance with the guidelines on good clinical practice and with the ethical standards for human experimentation established by the 1964 Declaration of Helsinki. All subjects gave informed consent before being included in the study. The study protocol was reviewed and approved by Institutional Review Board of Siriraj Hospital, Mahidol University, Bangkok, Thailand [Protocol No. 103/2553(EC3)]. Amniotic fluid was collected from the Department of Obstetrics and Gynecology, Faculty of Medicine Siriraj Hospital. Briefly, amniotic fluid samples were obtained by amniocentesis performed for routine fetal genetic diagnosis at 16–20 weeks of gestation, centrifuged at 2100RPM for 5 min, and the pellet retrieved for cell line establishment. AFSC were cultured in MEMα medium (Gibco, Invitrogen, Carlsbad, MA, USA) supplemented with 15% embryonic stem cell qualified fetal bovine serum (ES-FBS), 1% L-glutamine, 1% penicillin/streptomycin (Sigma-Aldrich, St. Louis, MO, USA), 20% Chang medium (Irvine Scientific, Santa Ana, CA, USA) at 37 °C with stable 5% CO_2_ in normoxic conditions^28^. Cell quantity, morphology and expression of Oct-4, CD29, CD34, CD44, CD45, CD73, CD90 and CD105 were evaluated. Chromosomal stability was assessed by karyotype (Supplementary data – Methods).

### Human cardiomyocytes (hCM) and umbilical cord endothelial cells (HUVEC) culture

All experiments were performed with the approval of the Institutional Biosafety Committee, National University of Singapore (NUS) under protocol 2018-00717. hCM (Passage 4, Promocell, Heidelberg, Germany) and HUVEC (Passage 4, Lonza, Basel, Switzerland) were cultured according to suppliers’ protocols for approximately 3–4 days in the recommended growth media (hCM—myocyte growth medium, Promocell, Heidelberg, Germany; HUVEC—endothelial growth medium EGM-2, Lonza, Basel Switzerland) and cell seeding density (hCM—10,000 cells/cm^2^; HUVEC—5,000 cells/cm^2^). For expansion, the cells were maintained in an incubator at 37 °C with stable 5% CO_2_ in normoxic conditions with two-daily medium change. hCM and HUVEC were washed with phosphate buffered saline buffer (PBS, Gibco, Carlsbad, CA, USA) or HEPES buffered saline solution (HBSS, Lonza, Basel, Switzerland) respectively, trypsinized and harvested according to the suppliers’ instructions. hCM and HUVEC were cultured in 96-well plates at the recommended cell seeding density (n = 5 for oxygen condition, cell culture media and individual time-points) in preparation for proliferation assays. Cells were maintained at 37 °C with stable 5% CO_2_ in normoxia or hypoxia. Cells were cultured in 100μL of (1) recommended growth media, (2) basal MEMα, (3) normoxic AFSC secretome (nAFSC-S) or 4) hypoxic AFSC secretome (hAFSC-S).

### Generation of amniotic fluid stem cells secretome (AFSC-S)

Three AFSC cell lines were randomly selected for the initial evaluation of cell morphology and expression of pluripotent and cell surface markers. The secretomes from these cell lines were individually analyzed for total protein. Cell morphology and surface marker expression were similar, as were total secretome proteins (0.304, 0.326 and 0.319 mg/ml respectively). Therefore, one AFSC line was randomly selected for the current study. To evaluate the optimal culture duration to maximize protein production and release, we thawed AFSC at passage 8 to culture at a cell seeding density of 8,500 cells/cm^2^ in AFSC growth media in normoxic and hypoxic oxygen conditions for 48 h to reach 80% confluence. Collected AFSC were washed twice with 1XPBS (Gibco, Carlsbad, CA, USA) and cultured in basal MEMα (Thermo Fisher Scientific, Waltham, MA, USA). AFSC-S was collected 6-hourly from 6 to 30 h (n = 3 per time-point in both oxygen conditions) and prepared such that each mL of collected volume represented one million AFSC. Collected nAFSC-S and hAFSC-S were filtered and stored at − 80 °C prior to use. AFSC were quantified following secretome harvest. Total protein was assessed in AFSC and AFSC-S in a Bradford assay with Coomassie Blue (Bradford assay, Bio-Rad, Hercules, CA, USA). Cell morphology was evaluated microscopically at all harvest time-points. AFSC-S were collected in biological triplicates for each parameter (normoxic and hypoxic conditions for each predetermined time-point), triplicates pooled, and the pooled samples normalized to one million cells by addition of basal MEMα prior to use in culture. The 30 h-hAFSC-S was standardized to one million cells by dilution with basal MEMα, then concentrated to 20X using centrifugal filter units (Amicron Ultra, Merck, Kenilworth, NJ, USA).

### Amniotic fluid stem cells secretome (AFSC-S) characterization

Proteomic analysis of AFSC-S was performed with an antibody-based magnetic beads immunoassay using selected preconfigured panels (Procartaplex, Cytokine/Chemokine/Growth Factor Convenience 45-Plex Human Panel 1, Thermo Fisher Scientific, Waltham, MA, USA) for multiplex protein quantification on the Luminex instrument platform (Luminex, Luminex Corporation, Austin, TX, USA). A selection of chemokines, cytokines and growth factors were evaluated (Table 1A). Briefly, prepared antibody magnetic beads and standards for 45 analytes were added to a 96-well plate. Thawed AFSC-S samples were incubated with antibody magnetic beads (120 min), washed, incubated with detection antibodies (120 min), washed, and Streptavidin-Phycoerithrin (SAPE) added for final incubation (5 min). All incubations were done on a shaker at 500RPM. Samples were then mixed with supplier’s reading buffer, and fluorescence recorded with Luminex 200 (Luminex Corporation, Austin, TX, USA). Analyte concentrations were normalized to one million AFSC. Mean concentrations of individual analytes (n = 3–8) at each time-point generated in either secretome are represented as a heatmap showing the temporal variation in analyte concentrations.

### Proliferation assay

In order to evaluate the number of viable cells in proliferation, we performed a colorimetric assay with a tetrazolium compound (Owen’s reagent) that cells bioreduce to form a colored formazan product. Briefly, cells were cultured in 96-well plate for 24 or 72 h in the selected oxygen conditions and culture media. MTS compound (CellTiter 96AQ_ueous_, Promega, Madison, WA, USA) was added directly to cell culture media in a volume of 20μL and cells incubated for 4 h. To produce cell proliferation curves in different culture conditions, absorbance was recorded using 490 nm and 630 nm wavelengths at 15 min, 60 min, 120 min, 180 min and 240 min for hCM, and 120 min and 240 min for HUVEC. Final absorbance values were normalized to cellular debris and blanks (630 nm). Proliferation fold-change was determined as the ratio of absorbance values of secretome to basal media cell cultures.

### Animal studies

We performed proof-of-concept experiments to evaluate the therapeutic potential of AFSC-S on cardiac ischemia in a mouse model of IR injury as described previously^8^. All animal experiments were approved and performed in compliance with the NUS Institutional Animal Care and Use Committee protocol R18-1452 and carried out in accordance with the National Advisory Committee for Laboratory Animal Research (NACLAR) Guidelines. The animal study conformed to the “Guide for the Care and Use of Laboratory Animals” published by the US National Academies funded by the National Institutes of Health (Guide for the Care and Use of Laboratory Animals, 8^th^ Edition, National Academies Press, Washington, USA, 2011, www.national-academies.org). Based on our proliferation results, we selected the hAFSC-S collected at 30 h (30 h-hAFSC-S) as the most effective for transfusion. Briefly, male C57BL/6 J mice (n = 11; mass 30 ± 2 g; age 12.4 ± 2.2 months; InVivos, Singapore) were anesthetized with a mixture of 0.5 mg/kg medetomidine (Domitor, Pfizer Animal Health, Exton, PA, USA), 5.0 mg/kg midazolam (Dormicum, Sciencelab, Dickinson, ND, USA) and 0.05 mg/kg fentanyl (Fentanyl Citrate Injection, Pfizer Pharmaceuticals Group, New York, NY, USA) via intraperitoneal (IP) injection, intubated and oxygenated on a rodent ventilator (Model 845 MiniVent Ventilator for Mice, Harvard Apparatus, Holliston, MA, USA). Lateral thoracotomy was performed, and mice were subjected to 30 min occlusion of the left anterior descending coronary artery (LAD) with Vicryl 6/0 (Johnson&Johnson, New Brunswick, NJ, USA) followed by reperfusion, mimicking the clinical setting of patients undergoing primary percutaneous coronary intervention. Mice were injected with 150μL of saline or 30 h-hAFSC-S via tail vein 5 min before reperfusion. The sham animal did not undergo any surgery and/or treatment. Animals recovered from anesthesia following subcutaneous injection of atipamezole (2.5 mg/kg, Antisedan, Pfizer Animal Health, Exton, PA, USA) and flumazenil (0.5 mg/kg, Flumazenil Injection, Sagent Pharmaceuticals, Schaumburg, IL, USA), with subcutaneous injection of buprenorphine (01.mg/kg, Buprenorphine Hydrochloride Injection, Hospira Inc., Lake Forest, IL, USA) for analgesia. Mice were euthanized 24 h post-surgery with an overdose of IP ketamine (225 mg/kg, Ketaset, Fort Dodge, IA, USA) and medetomidine (3 mg/kg, Domitor, Pfizer Animal Health, Exton, PA, USA).

### Histological evaluation of animal tissue samples

Hearts were harvested and divided into three groups. The first group of tissues was infused with Evan’s blue (Sigma-Aldrich, St. Louis, MO, USA), cross-sectioned in five planes and stained in 1% TTC (2,3,5-triphenyl tetrazolium chloride solution, Sigma-Aldrich, St.Louis, MO, USA). Heart sections were imaged with an inverted microscope for quantitative assessment of infarct size, presented as percentage of the infarct area (dead tissue) of area at risk (total ischemia-affected tissue). The second group of tissues was fixed in 10% neutral buffered formalin (NBF, Sigma-Aldrich, St. Louis, MO, USA), embedded in paraffin (Surgipath Paraplast Plus, Leica Biosystems, Nussloch, Germany) and sectioned at 4 μm for hematoxylin and eosin (H&E) and Masson’s trichome staining, and at 10 μm for Terminal deoxynucleotidyl transferase dUTP nick end labeling (TUNEL staining using TUNEL Assay Kit-HRP-DAP; Abcam, Cambridge UK), following standard procedures and manufacturer’s instructions. Nuclei were counterstained with hematoxylin. Immunohistochemistry (IHC) was performed with antibodies for proliferation (Ki-67, Thermo Fisher Scientific, Waltham, MA, USA), monocytes/granulocytes/neutrophils (Ly6G6D) and macrophages (MAC3, both from BD Pharmigen, San Diego, CA, USA); all antibodies were used in 1:100 concentration and sections counterstained with hematoxylin. Quantification of apoptotic cells was performed by randomly selecting three TUNEL-stained sections per sample, evaluating 8–10 microscopic fields per section and determining the percentage of TUNEL-positive nuclei over total nuclei in each field.

### Gene regulation evaluation in animal tissue samples

The remaining samples were dissected into infarct (I) and remote (R) areas, snap frozen in liquid nitrogen and processed for quantitative real-time polymerase chain reaction (qRT-PCR). Total RNA from all samples were extracted using TRIzol™ (Invitrogen, Carlsbad, MA, USA) and RNeasy Mini kit (Qiagen, Venlo, Netherlands) and transcribed to cDNA using Tetro cDNA Synthesis kit (Bioline, Eveleigh NSW, Australia). Primers (Supplementary Table 1) were used at a final concentration of 1 μM with 20 ng cDNA in a SYBR green master mix (Applied Biosystems, Foster City, CA, USA) per well in a 96-well plate for qRT-PCR analysis using ABI PRISM 7500 Fast Real-Time PCR System (Applied Biosystems, Waltham, MA, USA). Cycling conditions involved initial denaturation (95 °C, 10 min), 40 cycles of denaturation (95 °C, 15 s), annealing (60 °C, 30 s) and extension (72 °C, 1 min) and final extension at 72 °C for 10 min. β-Actin served as the normalization control and the sham sample served as the calibrator. The relative quantification was performed using the comparative CT (2−ΔΔCT) method. The qRT-PCR was performed for 35 cycles of initial denaturation at 95 °C for 10 min and 35 cycles of DNA denaturing at 95 °C for 30 s. The annealing temperatures used were 62 °C for interested genes and 57 °C for beta-actin.

### Imaging

Samples were imaged with an Olympus IX73 inverted microscope equipped with the IXplore Standard imaging system (Olympus, Shinjuku City, Tokyo, Japan). All acquisition parameters remained constant during the imaging process. Images were captured from each sample at 10x, 20x and 40x magnification and processed with Image J 1.52q (ImageJ, NIH, Bethesda, MD, USA, imagej.net) for analysis.

### Statistical evaluation

All results are represented as mean ± SD. Statistical evaluation of multiple groups was performed using one-way or two-way ANOVA with Tukey’s *post-hoc* test as appropriate. Animal data collected from TTC stained samples were evaluated by unpaired t-test. Statistical tests were considered significant when *p* < 0.05 (*p < 0.05, **p ≤ 0.01, ***p ≤ 0.001, ****p ≤ 0.0001). All statistical analysis was performed using QuickCalcs and GraphPad Prism (version 8.4.2., GraphPad Software, San Diego, CA, USA, graphpad.com).

### Ethics approval

The study was conducted in accordance with the guidelines on good clinical practice and with the ethical standards for human experimentation established by the 1964 Declaration of Helsinki. The study protocol was reviewed and approved by Institutional Review Board of Siriraj Hospital, Mahidol University, Bangkok, Thailand [Protocol No. 103/2553(EC3)]. All animal experiments were approved and performed in compliance with the NUS Institutional Animal Care and Use Committee protocol R18-1452 and carried out in accordance with the National Advisory Committee for Laboratory Animal Research (NACLAR) Guidelines. All experiments were performed with the approval of the Institutional Biosafety Committee, National University of Singapore (NUS) under protocol 2018-00717.

### Consent to participate and publish

All subjects gave informed consent before being included in the study.

## Supplementary Information

Supplementary Information.

## Data Availability

The data are available from the corresponding author on a reasonable request.
