# Supplementary material for: Hypoxia-induced amniotic fluid stem cell secretome augments cardiomyocyte proliferation and enhances cardioprotective effects under hypoxic-ischemic conditions
Source: Sci Rep. 2021 Jan 8;11:163. doi: 10.1038/s41598-020-80326-w (PMC7794288; doi:10.1038/s41598-020-80326-w)
Supplement: Supplementary file 1 — Supplementary Information. [file 41598_2020_80326_MOESM1_ESM.docx]

**Scientific Reports - Original Research**

**Supplementary Material.**

**Title: Hypoxia-induced amniotic fluid stem cell secretome augments cardiomyocyte proliferation and enhances cardioprotective effects under hypoxic-ischemic conditions**

Authors

Marek Kukumberg^1,2^, Tatsanee Phermthai^3^, Suparat Wichitwiengrat^3^, Xiaoyuan Wang^2,4^, Subramanian Arjunan^1^, Chong Suet Yen^2,4^, Chui Yee Fong^1^, Jiong-Wei Wang^2,4,5^, Rufaihah Abdul Jalil^2*^, Citra Nurfarah Zaini Mattar^1,6*^

1. Department of Obstetrics and Gynaecology, Yong Loo Lin School of Medicine, National University of Singapore, Singapore

2. Department of Surgery, Yong Loo Lin School of Medicine, National University of Singapore, Singapore

3. Stem Cell Research and Development for Medical Therapy Unit, Department of Obstetrics and Gynecology, Faculty of Medicine Siriraj Hospital, Mahidol University, Bangkok, Thailand

4. Cardiovascular Research Institute, National University Heart Centre Singapore, Singapore

5. Department of Physiology, Yong Loo Lin School of Medicine, National University of Singapore, Singapore

6. Department of Obstetrics and Gynaecology, National University Health Systems, Singapore

* Joint senior authors

Corresponding author: Dr Citra NZ Mattar, Associate Professor, Experimental Fetal Medicine Group, Department of Obstetrics and Gynaecology, Yong Loo Lin School of Medicine, National University of Singapore, Singapore 119228, Telephone: +65-67722672, [citramattar@nus.edu.sg](mailto:citramattar@nus.edu.sg)

**Supplementary Material and Methods**

**Amniotic fluid stem cells proliferation analysis**

The amniotic fluid stem cells (AFSC) proliferation capacity was evaluated by population doubling time (PDT) at each subculture passage. The PDT was determined by the following equation: [log(2)T/log(Y) - log(X)], where T is the number of days for cell expansion at each passage, X is the seeded cell number, and Y is the number of harvested cells.

**Characteristics analysis**

For phenotypic analysis, AFSC from P5 culture at 70% confluence was detached and washed with 1XPBS. The cells were stained with fluorescein isothiocyanate- and phycoerythrin-conjugated monoclonal antibodies against CD29, CD34, CD45, CD73 CD90, and CD105 (eBioscience, San Diego, CA, USA) for 15 min in the dark. After PBS washing, the AFSC were fixed with 1% paraformaldehyde (Merck, Darmstadt, Germany) at room temperature. AFSC phenotypic analysis was performed using a Becton Dickinson flow cytometer (Becton Dickinson, Franklin Lakes, NJ, USA).

**Chromosome analysis**

AFSC was treated with colchicine and incubated at 37°C for 3 h. The AFSC were harvested using 0.25% trypsin-EDTA and transferred to a centrifuged tube containing culture medium. Samples were centrifuged at 1,800 rpm for 5 min and incubated with 0.075 M KCl at 37°C for 20 min. Chromosomes were fixed with precooled mixture of methanol and acetic acid at a proportion of 3:1. Fixed cells were delocalized and spread onto glass slides. The slides were stained using GTG banding (GTG: G-bands by trypsin using Giemsa staining) for investigation of stained bands on chromosome.

**RNA extraction and quantitative real-time polymerase chain reaction (qRT-PCR) for gene expression analysis**

AFSC was extracted for total cellular RNA using Trizol (Invitrogen, Carlsbad, CA, USA) according to the manufacturer’s instructions. The purity and quantity of the isolated RNA were evaluated using Nanophotometer version 2.0 (Implen, Inc., Munich, Germany). Individual RNA samples were synthesized into complementary DNA (cDNA) using a RevertAid First-Strand cDNA Synthesis Kit (Fermentas, Vilnius, Lithuania). The cDNA was applied for gene expression experiment.

The primers used in qRT-PCR are as follows:

Oct-4 (247 bp) sense, 5′-CGTGAAGCTGGAGAAGGAGAAGCTG-3′, and antisense, 5′-CAAGGGCCGCAGCTTACACATGTTC-3′

Col 2 (79 bp) sense, 5′-GGCAATAGCAGGTTCACGTACA-3′, and antisense, 5′-CGATAACAGTCTTGCCCCACTT-3′

beta-Actin (107 bp) sense, 5′-ATGTGGCCGAGGACTTTGATT-3′, and antisense, 5′-AGTGGGGTGGCTTTTAGGATG-3′

The cDNA was amplified in qRT-PCR reaction mixtures containing cDNA template, 0.2 mmol/L of deoxy nucleotide triphosphate, 1 U of Taq DNA polymerase and 0.2 pmol/L of each forward and reverse primers. The qRT-PCR was performed for 35 cycles of initial denaturation at 95°C for 10 min and 35 cycles of DNA denaturing at 95°C for 30 seconds, annealing for 45 seconds, extension at 72°C for 1 min and final extension at 72°C for 10 min. The annealing temperatures used were 62°C for the genes of interest and 57°C for beta-actin. Analysis of Collagen type II gene was performed using qRT-PCR.

**Supplementary table 1: Primers used for qRT-PCR gene expression analysis of animal tissue samples**

| Gene | Gene Symbol | Gene ID | Ref Seq ID |
| --- | --- | --- | --- |
| Angiopoietin-like protein 1 | ANGPTL1 | 11600 | NM_009640 |
| Angiopoietin 2 | ANGPTL2 | 11731 | NM_007449 |
| Vascular endothelial growth factor | VEGF | 22339 | NM_001025257 |
| Fibroblast growth factor | FGF | 14173 | NM_008006 |
| Hepatocyte growth factor | HGF | 15234 | NM_010427 |
| Transforming growth factor-beta | TGF-β | 21808 | NM_009367 |
| Interleukin 1 | IL-1 | 16175 | NM_010554 |
| Beta-actin | Β-ACTIN | 11461 | NM_007393 |
